# Editorial

**Published:** 1866-01

**Authors:** 


### Editorial.

CHICAGO MEDICAL SOCIETY.—*Discussion on Diphtheritic Croup, or Diphtheria involving the Larynx.—Lime-Water Inhalation.*—At a recent meeting of the Chicago Medical Society, while the subject of diphtheria as it invades the larynx was under discussion, Dr. J. P. ROSS stated that he had recently tried the inhalation of the vapor of lime-water, as suggested by some foreign writer:—In one very strongly-marked case, in which the obstruction of the larynx was so great that the child was not expected to live more than a very few hours, the inhalation soon produced some relief, and in a few hours a considerable quantity of detached and broken shreds of diphtheritic membrane was discharged, and the child ultimately recovered. In another case, however, the relief afforded by the inhalation was only temporary, the patient dying in a few hours. If the lime-water vapor merely acts as a solvent of the diphtheritic exudation upon the surface of the larynx and fauces, it must of necessity often fail to prevent a fatal termination in this form of diphtheria, because the dyspnœa, in many cases, arises more from the infiltration into, and consequent tumefaction of, the sub-mucous tissue at the base of the epiglottis and within the larynx, than from the amount of exudation upon the mucous surface. Still, so far as patients laboring under this much dreaded disease can be made to inhale aqueous vapor impregnated with alkalies or alkaline earths, it will, undoubtedly, aid materially in disintegrating the diphtheritic exudations, and, thereby, prove a valuable aid to other appropriate treatment. But it must not be forgotten that diphtheria is not a mere local disease of the larynx or fauces, but involves the whole system, depressing the vital properties and impairing the quality of the blood. Consequently, mere local applications of any kind cannot meet all the plain indications for treatment.

---

Illinois State Medical Society.—As the next annual meeting of the American Medical Association is to be held in Baltimore on the first Tuesday in May next, it will be necessary to hold the next meeting of our State Society on the first Tuesday in June. We hope the Committee of Arrangements at Decatur will remember this. As this brings the meeting of the State Society one month after that of the American Medical Association, the delegates to the latter, appointed last year at Bloomington, will hold their appointments for the coming meeting in Baltimore also.

The names of the delegates are as follows, viz.:—A. L. McArthur, M.D., of Joliet; L. T. Hewins, M.D., of Loda; J. W. Redden, M.D., of Shawneetown; J. M. Steele, M.D., of Grandview; J. S. Jewell, M.D., of Chicago; H. Noble, M.D., of Heyworth; T. F. Worrell, M.D., of Bloomington; E. L. Holmes, M.D., of Chicago; J. W. Freer, M.D., of Chicago; E. W. Moore, M.D., of Decatur; A. Niles, M.D., of Quincy; A. W. Heise, M.D., of Joliet; R. E. McVey, M.D., of Waverly; L. Clark, M.D., of Rockford; H. C. Luce, M.D., of Bloomington; D. Prince, M.D., of Jacksonville; D. Brainard, M.D., of

Chicago; W. A. Elder, M.D., of Bloomington; J. Brown, M.D., of Decatur; N. Wright, M.D., of Chatham.

We publish the names of the delegates, and call the attention of the Society to the change in the time of meeting thus early, for two purposes:—First, that all parties shall fully understand that the order of meeting for the state and national organizations for this year is reversed; the American Medical Association meeting at Baltimore, on the first Tuesday in May, and the Illinois State Medical Society, at Decatur, on the first Tuesday in June.

Second, that the delegates may have ample time to make their preparations to attend the meeting of the Association; and such of them as know they cannot attend, should inform the Secretary early, that substitutes may be selected.

---

COOK COUNTY HOSPITAL—The Board of Supervisors of this county, under whose care is placed the interests of the poor of this city, has finally completed the arrangements for opening a public hospital for the sick poor of the city and county. It will occupy the building well known as the *City Hospital*, between 18th and 19th Streets. The following are announced as constituting the medical and surgical staff of the hospital, viz.:—*Attending Physicians*, THOMAS BEVAN, M.D., J. P. ROSS, M.D., and H. W. JONES, M.D. *Attending Surgeons*, GEO. K. AMERMAN, M.D., R. A. BOGUE, M.D., and CHAS. G. SMITH, M.D. *Consulting Physicians*, H. A. JOHNSON, M.D. and R. C. HAMILL, M.D. *Consulting Surgeons*, J. W. FREER, M.D. and WM. WAGNER, M.D. *Pathologist*, HENRY M. LYMAN, M.D.

These are all excellent appointments, and we hope such regulations will be adopted by the Board of Supervisors as will prevent their being disturbed by the results of each annually recurring political election.

---

REVIVAL OF MEDICAL PERIODICALS IN THE SOUTH.—In our last issue, we noticed the promised appearance of a medical journal in Richmond, Va., and at Memphis, Tenn. Since then we have received notices that the *New Orleans Medical and*

*Surgical Journal* will be revived under the editorial management of Dr. BENNETT DOWLER, well known to the profession as an able writer; and that the publication of medical journals will also be resumed at Atlanta, Ga., and Charleston, S. C. We are also informed that a new medical periodical is about to make its appearance in New York City, to be published by WM. WOOD & Co., and edited by Dr. SCHRADY.

---

OBITUARY NOTICE.—Pursuant to a call, the members of the medical profession of the City of Rockford met on the 21st ult., and unanimously adopted the following preamble and resolutions:—

*Whereas*, By the dispensation of Providence, Dr. WILLIAM LYMAN has been called from time to eternity; therefore,

*Resolved*, That, by the death of Dr. LYMAN, the medical profession has been bereft of one of its most devoted and noble members; society, of a true friend and benefactor; his family, of a loving and large-hearted protector; and we, his professional associates, of a friend who, by his uniform kindness of heart and geniality of manner, has won our esteem and respect.

*Resolved*, That we extend to the family of the deceased, our sympathy for their bereavement, and that we attend the funeral in a body.

*Resolved*, That a copy of these resolutions be transmitted, by the Secretary, to the bereaved family, and copies for publication, to the *Chicago Medical Journal*, CHICAGO MEDICAL EXAMINER, and the *Rockford Register*.

G. W. ROHR, *Secretary*. HENRY STRONG, M.D., *Chairman*.

---

PROF. DANIEL BRAINARD.—This well-known surgeon, who has occupied the Chair of Surgery in Rush Medical College, ever since the College was organized, took his departure for Europe a few days since. His family had preceded him a few months; and it is understood that they intend to remain in Europe several years.

---

SANITARY SCIENCE.—In an interesting introductory lecture on this subject, Dr. A. B. PALMER, Professor of Pathology and Practical Medicine, in the Medical Department of the University of Michigan, gives the following encouraging statistics, in relation to the effects of sanitary improvements on health and mortality:—

In the whole of England and Wales a registration of births, marriages, and deaths is now, and has for a considerable time, been carefully, and, in the main, accurately kept; and in several municipalities, such records have been kept for a long period. From these, we learn that with the advancement of science and civilization, the annual percentage of mortality has gradually diminished, and at the present period the chances of life are better than ever before. Astonishing changes in particular localities have been effected by the removal of nuisances, and by improvements in drainage, in pavements, and in the construction of dwellings and the like—these improvements more particularly affecting the towns and the poor. Now, whenever in any locality the death rate rises much above the average, as ascertained by the reports sent to the Register-General's Office in London, enquiry is made into the cause, and when discovered, as it almost always is, measures are taken for its removal, and the death-rate falls to its usual level.

The following table from McCulloch's Statistics of the British Empire will show the progressive diminution of mortality for the last two centuries in the City of London:—

| Years. | Amount of Mortality. Per Cent. |
|---|---|
| 1660—35 | 8.000 |
| 1728—57 | 5.200 |
| 1771—80 | 5.000 |
| 1801—10 | 2.920 |
| 1831—35 Cholera years, | 3.200 |
| 1831—41 | 2.522 |
| 1851— | 2.340 |

Another table from the same work, shows the progress made during the last century in the management of children. The percentage of all the births of children dying before reaching the fifth year, was as follows:—

| Years. | Percentage of deaths under five years. |
|---|---|
| 1730—49 | 74.5 |
| 1750—69 | 63.0 |
| 1770—89 | 51.5 |
| 1790—1809 | 41.5 |
| 1810—29 | 31.8 |
| 1851 | 25.8 |

Similar changes have occurred in other countries. In France, for instance, at the close of the last century, the mean average duration of human life was less than twenty-nine years, whereas a dozen years ago it was thirty-three.

About one hundred years ago, in the imperfectly ventilated and badly managed workhouses of London, twenty-three out of every twenty-four children, born and retained in those establishments, died before attaining the age of one year. Such terrible mortality attracted the attention of Parliament; the causes were enquired into, and, by an act of that body, directing certain reforms, the mortality was immediately reduced from 95.83 per cent., to 13 per cent.—from 2,600 to 450 in a single year, thus saving 2,150 lives in those establishments alone. The present death-rate, during the first year of life, in all England, is 16.559 per cent. This act of Parliament, and the great care in management which immediately followed, reduced the mortality in the poorhouses much below the present average rate in the whole country. Human agencies seem thus to have something to do in influencing the issues of life and death.

At the present time, in the more rural districts of England, where the children have purer air and better attention, of all under five years, the annual death-rate is about 4 per cent.—or 40 in 1000; while in East London, Sheffield, Coventry, Leeds, and other similar places, it is over 10 per cent. In Manchester, a crowded manufacturing city, it is 11.7 per cent., and in Liverpool, a crowded, filthy seaport, it is 13.198 per cent., or 131.98 in 1000.

The mortuary statistics of our own country have been but imperfectly kept. In many of the states—in our own state, though so far advanced in the means of education, and in so many other respects, there is no general law on the subject of registration, and we have no authoritative means of knowing the proportions that are living or dying. This is a reproach, which, it is to be hoped, will soon be wiped away. In most of the cities throughout the country, records are kept, and we find these records showing, among others, the following facts in 1863:—

| Place. | No. of deaths to each thousand of population. |
|---|---|
| New York, | 27.0 |
| Philadelphia, | 23.1 |
| Boston, | 24.0 |
| Newark, N.J., | 22.9 |
| Providence, R.I., | 22.7 |
| Hartford, Conn., | 18.2 |

In most rural districts the percentage of deaths is still smaller, and might be much further reduced if all the hygienic laws were obeyed. In New York, about 25,000 die annually. If the laws of health were obeyed as well, and no better, than in many rural districts, the deaths would not be more than 15,000. It thus appears that, at least, there are over 10,000 unnecessary deaths in that city. To every death there is estimated to be twenty cases of sickness, and for these 10,000 unnecessary deaths, there are 200,000 cases of unnecessary sickness. Each case of sickness is estimated to cost on an average, in loss of time, in medical attendance, and nursing, about $50. Now, the 200,000 cases of preventable sickness, multiplied by the number of dollars each costs, would give us $10,000,000 as the result. This vast sum of ten millions of dollars is annually spent in this one city for unnecessary sickness. But this is almost unworthy of mention in comparison with the bodily and mental suffering thus needlessly endured.

In whatever aspect this subject is viewed, its magnitude and importance surprise us; and the indifference with which it is treated by most men surprises us more.

Notwithstanding all these facts—the changes in the bills of mortality effected by changes in the habits of the people, and the conditions of their surroundings—permanent changes from 80 to 23 per thousand in a vast and still growing city—notwithstanding an act of Parliament reduced the mortality among the innocents in the poorhouses of London from 2,600 to 450, saving 2,150 lives in a single year, yet there is a sort of vague impression in the minds of many, that death, if not sickness, is a matter of special providence—a thing over which human agency has no control. If this be so, Gentlemen, our "occupation's gone." You may as well go home and let this department of the University be closed. These walls now devoted to science and an active humanity, should be given up to the teaching of a paralyzing fatalistic philosophy. Arabia could, doubtless, furnish a Mohammedan professor who would teach us to fold our hands in indifference, and exclaim, "Allah ruleth!" The Bible, however, teaches that, "whatsoever a man soweth that shall he also reap;" and the freedom and efficiency of human agency are everywhere taught. The fatalistic theory is as false in its application to this as to other subjects.

To the sentiments embraced in the following paragraph, from the same address, we cordially assent:—

Now, Gentlemen, of the importance of this subject of sanitary science—of a knowledge of its principles and a practical obedience to its precepts, no one can doubt. That this knowledge should be possessed by non-professional persons in order that its precepts be generally understood and observed, is equally evident; and it is also too apparent to require enforcing, that to professional men—to physicians, the community must chiefly look for this knowledge. It will be for you in your several stations and communities, after first obtaining it, to spread abroad your light. You should urge these subjects as branches of popular education. You should encourage and aid in the introduction of articles upon them into our periodical literature. The newspaper and the magazine—those great educators of the people, should be made channels through which this knowledge should flow abroad; and should there at any time occur within your sphere of observation any unusual sickness and mortality, it will be your duty as *guardians* of the *public health* and *teachers* of the people, to investigate the causes of such sickness with minuteness and faithfulness; and it will be in opposition to your duty to hide under a bushel your conclusions and the facts upon which they are based, for any temporary effect which the statement of such facts and conclusions may have upon either your own feelings and passing interests, or those of others. You cannot be too careful in arriving at the facts, or too rigid in your deductions from them; but when the facts have been ascertained beyond a reasonable doubt, and when your logic is without a perceptible flaw, place your light upon a candlestick, that it may yield the illumination so much needed.

---

Foreign Correspondence.—We copy the following interesting letter from the *St. Louis Medical and Surgical Journal* for November and December:—

LETTER FROM PROFESSOR CHAS. A. POPE, M.D.

Paris, August 27, 1865.

Messrs. Editors: It has been my pleasure, for some months past, to attend weekly, at the Hospital Necker, the cliniques of M. Civiale. During this time I have seen him perform a large number of his usual operations, such as urethrotomy, lithrotripsy, and lithotomy. In the crushing of calculi he is still unrivalled. The extreme care with which he examines his cases, and the wonderful ease, delicacy, and precision which characterize his manipulations, have not diminished with his advancing years. Compliments in this respect, however, he generally declines, preferring that his great skill should be shared by all surgeons. His wish is to see lithrotripsy practiced by the whole profession, and not confined to a few. Forty years' observation and experience in an extensive and special practice could not fail to render him thoroughly proficient in all that concerns the diseases of the urinary organs. It must be recollected that in the diagnosis of these we are deprived of the use of sight, and are guided by the touch alone. Nor is the touch direct, for it is exercised through the medium of a long, metallic instrument. One's admiration, therefore, is enhanced to see with what accuracy he appreciates the various conditions of the urethra and bladder, the presence or absence of calculi, whether free or encysted, their size, shape, hardness, and number, as well as the various tumors and polypi, their narrow or broad base, and, in short, all that concerns the pathology of these important organs. Yet, notwithstanding his ability, Civiale is always modest, and deprecates the rashness and presumption of those who claim to be absolute in their diagnosis and treatment of these difficult and often obscure complaints. The method and instruments are the same which he has employed for the last thirty years. If there be one quality which characterizes him more than another, it is gentleness. This is his great watchword. He never inflicts useless pain, never fatigues his patient, by prolonged operations, and always properly prepares them for the use of his instruments, which seem to glide of their own accord, and are of small size to admit of freer play. A favorite remark with him is, always to give the urethra time to swallow the instruments, the wisdom of which is apparent to all who are familiar with such operations. His lithrotripsycal operations are almost perfect.

In nodulated strictures he still uses his own urethrotome, which cuts, as you know, from behind forwards, or from the bladder to the penis. Sometimes he incises the urethra for seven inches in extent; but, generally, he limits the incision to the mere nodosity. He prefers to act on the posterior wall of the urethra, whilst Maissonneuve almost always cuts anteriorly. Both are careful to introduce immediately an elastic catheter, which is retained for thirty-six or forty-eight hours.

In lithrotripsy I have said that Civiale is unrivalled; but in lithotomy he is by no means expert. Blunt instruments along natural routes he thoroughly understands; but cutting instruments are out of place in his hands. My recollection of him would have ever been one of unalloyed pleasure and admiration, but for an unfortunate case of lithotomy which I not long since witnessed, in which he but too plainly evinced his want of surgical skill.

The patient was a man about fifty-five years of age—the stone being of rather large size. No chloroform was used, and, during the protracted operation, his sufferings were very great. A straight incision was made in the median line, in front of the anus. The staff having been reached, a double *lithotome cache* was pushed into the bladder, and, the blades having been made to project, the instrument was withdrawn. With the common lithotomy forceps an attempt was made to extract the stone, but failing, resort was had to a complex apparatus for crushing, or rather disrupting, the foreign body. This consisted of a large forceps, whose strong handles were forcibly approximated by a vice, through which and the joint of the forceps a perforating instrument like a gimblet and worked by a screw was introduced against the included stone with the view of boring into it, and thus causing its disruption. After much time, labor, and suffering, the operator succeeded in breaking off and removing a portion of calculus, more than an hour having been consumed in ineffectual attempts at extraction, during which both the surgeon and patient were thoroughly exhausted; the latter was removed to his bed with the promise that the remainder of the stone would be taken from him in a few days. He died, however, on the second day, and the stone was removed after death. It constituted about two-thirds of the whole mass. When entire, it was ovoidal in shape, and would have measured about 2¾ inches in length by 2¼ inches in thickness. I have myself successfully removed a larger stone from a much smaller patient by the single lateral incision.

A worse operation I scarcely ever witnessed. Its plan was defective, as the direction of the outer and inner incisions were at right angles to each other, and its execution was unskilful. The surgeon did not give the ordinary forceps a fair trial; and if, as he said, the stone was in the bas-fond of the bladder, which closely embraced it, why did he not introduce his finger into the rectum so as to facilitate the seizure? He seemed more intent in applying a sort of perineal lithrotripsy through the wound; but, whenever this becomes necessary, it were preferable to resort at once to hypogastric lithotomy.

For his own reputation, and the good of his patients, it is to he hoped that the able lithrotriptor will not often attempt the knife, but in cases requiring its use that he will hand them over to some more skilful confrere. Civiale seems to be about seventy years old. He is followed by a small class of from ten to fifteen students, the majority of whom are foreigners. He is said to be very wealthy, and pays, I have been told, six thousand francs a-year for his service at the Hospital Necker.

At this same hospital I have occasionally seen the application of the *endoscope* in the hands of its inventor, M. Désormeaux. Although ingenious, it does not strike me as being of much practical value. Thus far, at least, its revelations have not enabled us to treat the diseases of the urethra and bladder with any greater success so that, at present, it may be regarded as more curious than useful.

By means of a perforated mirror, placed obliquely and arranged precisely like Hutchinson's car speculum, the light of a lamp is projected through a silver tube into the urethra, the changes of whose walls we may plainly see, as also the margin of the prostate and the interior of the bladder. This last is accomplished by an angular catheter, having a small, glass window at the convexity or apex of the angle, which, whilst permitting vision, prevents the flow of urine which would obscure it. In the normal state the prostatic margin presents a crescentic shape, with the convexity posteriorly or inferiorly; if hypertrophied, this convexity is forward or superiorly. The inner surface of the bladder can be plainly seen, and the vascular ramifications on a whitish ground easily distinguished.

M. Désormeaux has several times observed encysted calculi and different fungous growths contained in the living bladder.

More than twenty years ago the idea of looking into the bladder was entertained by Segalas and others, but fell into neglect until a few years ago, when M. Désormeaux revived and accomplished it. This gentleman certainly deserves much credit for his preserving efforts in this direction; and it is to be hoped that, through the instrumentality of his urethroscope or endoscope—for he applies it to the bladder, and rectum also —valuable additions to our means of diagnosis and treatment may yet be effected. His pleasant manner and polite attention to strangers are quite remarkable, and enlist all such visitors in his favor.

Among the subjects which have greatly interested me during my sojourn in Paris, is one which, although largely medical, has yet an important surgical bearing. I allude to the application of electricity in the treatment of disease.

M. Duchesne (de Boulogne), so well known be his writings on this subject, is always pleased to receive visitors at his consultations, where his numerous patients afford a fine field for study and observation. His collection of casts, photographs and specimens, microscopical and pathological, is very extensive and unique. No other, perhaps, has so well investigated muscular action. From the study of the muscles he was naturally led to that of the nerves which influence them. The various changes which take place in both the brain and spinal marrow, corresponding to the different kinds of palsey, are beautifully shown under the microscope, the views of which have been most successfully photographed. It was, I believe, M. Duchesne who first discovered that the muscles, which failed to respond to the influence of the will, were entirely susceptible to the influence of the electric irritation, and vice versa. This view, at least in the first proposition, has been confirmed by our own surgeons in gun-shot injuries of nerves, as also by the experiments on animals which I have had the pleasure of witnessing at the *College de France*, by M. Claude Bernard, on the action of the Woorara poison.

In paralysis, the result of cerebral lesions, the muscular tissue, although atrophied from the want of use, undergoes but little other alteration, whilst in that dependent on changes in the spinal marrow, the fatty degeneration of muscles sooner or later ensues, and sometimes with great rapidity. The muscles which have undergone this fatty transformation are not susceptible to the electric influence. The larger number of cases of club-foot and club-hand are dependent on this latter condition, the transformation affecting one or more of the muscles of the limbs, so that, in these instances, tenotomy seldom does much good. If the whole tissue of a muscle have become transformed into fat, nothing can restore it. The loss, in this way, of the single *tibialis anticus*, M. Duchesne considers as more serious than that of all the other muscles controlling the motions of the foot. When thus singly affected, there is a resulting *pes equinus*, with eversion of the foot by the peronei muscles, and this deformity increasing with the growth of the patient, he at length walks on the inner ankle. Were, however, the effect general, *i.e.*, embracing all the muscles of the leg, an apparatus can be worn, which, giving support to the joint, also keeps the foot in proper position, and thus diminishes both the lameness and the deformity.

A case of contracted little finger was recently presented at the clinique of Nélaton, which M. Duchesne well showed to depend on the tendon of the flexer profundus. On the opposite arm of the patient the electric current, when applied to the superficial flexor, caused the fingers to bend only at the metacarpo-phalangeal articulation; whilst its application to the inner side of the arm, over the deep flexor, caused the additional bending of all the joints of the fingers themselves. The contrast in the flexion was very marked. M. Nélaton divided the tendon of the profundus in the palm of the hand, at its cubital margin.

On another occasion, at the same clinique, in attempting to show, on a freshly amputated leg, the accuracy with which any single muscle may be made to contract under the electric stimules, M. Duchesne was not a little disconcerted at his incomplete success, which, at the moment, he could not explain. The cause of his failure was discovered afterwards to have been a pathological agglutination of the muscles, which prevented their separate action.

In a case of "painful flat-foot," Nélaton, contrary to the opinion of M. Duchesne, divided the tendon of the long peroneus. The pain ceased for a time, that is, as long as the patient was quiet in bed. On using the limb, however, or on standing for some time, the pain returned, the patient stating that her condition was not at all improved. Having waited for the reparation of the divided tendon, M. Duchesne applied his battery, the woman avowing her improvement after each application. He states confidently that in a short time she will be completely relieved.

In these cases it is the inefficiency of the long peroneus which causes the flat-foot, being a sort of valgus, and the pain is owing to the unaccustomed pressure of the tarsal bones at the upper and outer part of the foot, where it is invariably found, at the depression of the cuboid bone and the astragalus. The arch of the foot also being destroyed, there results a painful compression of the plantar nerves.

There is, likewise, a pastry cook who had suffered in this way for many years. Although twenty years old, his pain has been entirely relieved by the electric current, and he is able to stand all day. Under its repeated use, also, there is an evident commencement of an arch to his foot.

There is a variety of paralysis, a marked specimen of which was exhibited in a boy, that M. Duchesne calls "*paralysie hypertrophique!* The muscles of the legs, though weak, are overdeveloped in size. The upper and lower limbs of the paraplegic patient seemed to belong to different individuals, so great was their disparity. Several photographic pictures of other patients exhibited the same variety of this unusual paralysis. In its cure, the hypertrophied muscles become reduced to their normal size.

A woman consulted M. Duchesne, complaining of intense though transitory pains, shooting down the right thigh, and coming on at intervals for two or three days, and then disappearing for several months, to recur again in the same way. The arm of the same side was also somewhat effected. He remarked that such cases, in their incipiency, were very commonly mistaken for and treated as rheumatism or neuralgia, when, in reality, they were the precursory symptoms of a most grave affection. The case was one of what is called "*ataxie locomotrice progressive.*" The patient already presents other confirmatory symptoms in the ambliopia, dilatation of the pupil, and changes in the optic papilla, with inability to walk steadily with the eyes closed, all showing the cerebral trouble. The nitrate of silver has recently been much vaunted in the treatment of this affection; but as there was a slight syphilitic taint, M. Duchesne entertained the hope of relieving his patient by means of the iodide of potassium and electricity, for although the tactile sensibility of the limb was impared, its electric sensibility and contractility were not.

M. Duchesne is now publishing a new work on his favorite subject. It is a field which, in a sense, he has made his own; and the patience and enlightened zeal with which he has cultivated it are deserving of all praise. It is to be hoped that his researches will lead to still further discoveries, and confer yet more important benefits on the profession and humanity.

Yours very truly,
CHAS. A. POPE.

---

INVESTIGATIONS UNDERTAKEN TO PROVE THAT THE ELECTRIC CONDITION OF MINERAL WATERS IS THE PRINCIPAL CAUSE OF THEIR ACTIVITY.—M. Scoutetten has of late worked very hard to prove the above proposition, and has published several accounts of his researches in the French medical papers. To give these researches some authenticity, M. Scoutetten repeated his experiments, a short time ago, at Mont Dore, a watering-place in Auvergne, under the eye of a distinguished medical committee, whose report is inserted in *L'Union Medicale* of the 25th of July. The author states, in his introduction, that "his researches throw a new light on the question; they prove that the waters, when emerging from the soil, are in an exceptionally active condition, that a chemical action productive of electrical phenomena is going on; and that to this cause the general effects of mineral waters are to be attributed." The experiments undertaken before the committee alluded to above, have proved—1. That the platinum electrodes, placed in ordinary water contained in a glass or china vessel, are not affected by even a trace of dynamic electricity; and that the needle of Nobili's galvanometer remained motionless. 2. That the same experiment, performed with mineral water immediately caused a considerable deviation of the needle. 3. That the same mineral water was examined in a like manner at various periods after collecting the water at the spring, and at different degrees of temperature, it being found that the elevation of the temperature sensibly increases the electrical manifestations; that the latter becomes weaker, on the contrary, the later the water is examined after emerging from the spring, which peculiarity is easily explained by the diminution and subsequent cessation of chemical action. Another experiment has shown that the immersion of only a portion of the human frame in mineral water is sufficient instantly to give rise to electrical phenomena, which are rendered manifest by the deviation of the needle. This important fact explains the exciting qualities of mineral waters, which qualities may be so powerful as to induce a feverish state in the individual acted upon. This property exists in all mineral waters, but in different degrees, regulated by the activity of the chemical combinations. This electrical action cures diseases by strengthening weakened organisms, which diseases are apparently widely different, but are nevertheless merely local manifestations of a general morbid state. It should be added that several experiments were undertaken with gold leaves, so as to prove that statical electricity does not exist in mineral waters. The latter have also been tried mixed with milk or syrup, and it was found that this mixture sensibly lessens the active properties of the waters.—*London Lancet.*

---

Chronic Farcy in Man.—A case is published in a French journal of a blacksmith who used to sleep in the stables. One of the horses was taken ill and died, without any symptoms of glanders, but affected with an extremely fœted diarrhœa. Soon afterwards the man sickened and died exhausted in hospital, with a great number of tumors which had formed in different parts of the body. One of these suppurated, and the pus was inoculated in various ways on an old horse and a donkey. Both died a few days after—the first with signs of chronic farcy, the second with those of acute glanders. The three autopsies revealed extensive deposits of softish and small tumors in the lungs.—*London Lancet.*

